# Thiazole orange-carboplatin triplex-forming oligonucleotide (TFO) combination probes enhance targeted DNA crosslinking†

**DOI:** 10.1039/d3md00548h

**Published:** 2023-10-31

**Authors:** Joseph Hennessy, Piotr Klimkowski, Daniel Singleton, Alex Gibney, Malou Coche, Nicholas P. Farrell, Afaf H. El-Sagheer, Tom Brown, Andrew Kellett

**Affiliations:** a SSPC, The Science Foundation Ireland Research Centre for Pharmaceuticals, School of Chemical Sciences, Dublin City University Glasnevin Dublin 9 Ireland andrew.kellett@dcu.ie; b Chemistry Research Laboratory, University of Oxford 12 Mansfield Road Oxford OX1 3TA UK; c ATDBio Ltd., School of Chemistry, University of Southampton Southampton SO17 1BJ UK; d Department of Chemistry, Virginia Commonwealth University Richmond VA 23284-2006 USA; e School of Chemistry, University of Southampton Southampton SO17 1BJ UK; f Department of Science and Mathematics, Faculty of Petroleum and Mining, Engineering, Suez University Suez 43721 Egypt

## Abstract

We report a new class of carboplatin-TFO hybrid that incorporates a bifunctional alkyne–amine nucleobase monomer called AP-C3-dT that enables dual ‘click’ platinum(ii) drug conjugation and thiazole orange fluorophore coupling. Thiazole orange enhances the binding of Pt(ii)-TFO hybrids and provides an intrinsic method for monitoring triplex formation. These hybrid constructs possess increased stabilisation and crosslinking properties in comparison to earlier Pt(ii)-TFOs, and demonstrate sequence-specific binding at neutral pH.

## Abstract 2

We report a new class of carboplatin-TFO hybrid that incorporates a bifunctional alkyne–amine nucleobase monomer called AP-C3-dT that enables dual ‘click’ platinum(ii) drug conjugation and thiazole orange fluorophore coupling.

## Graphical abstract

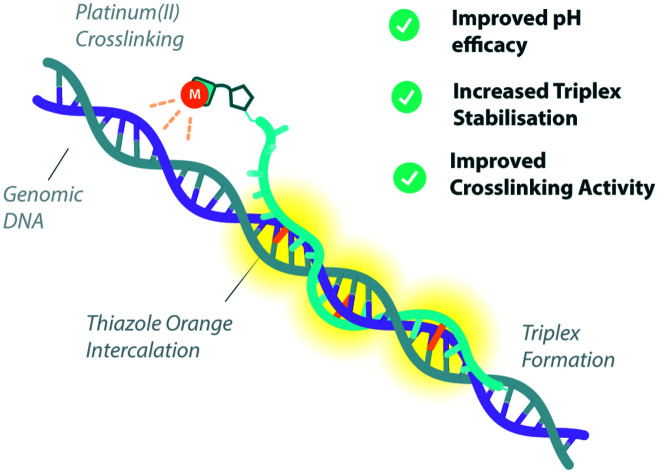

## Introduction

**Fig. 1:**
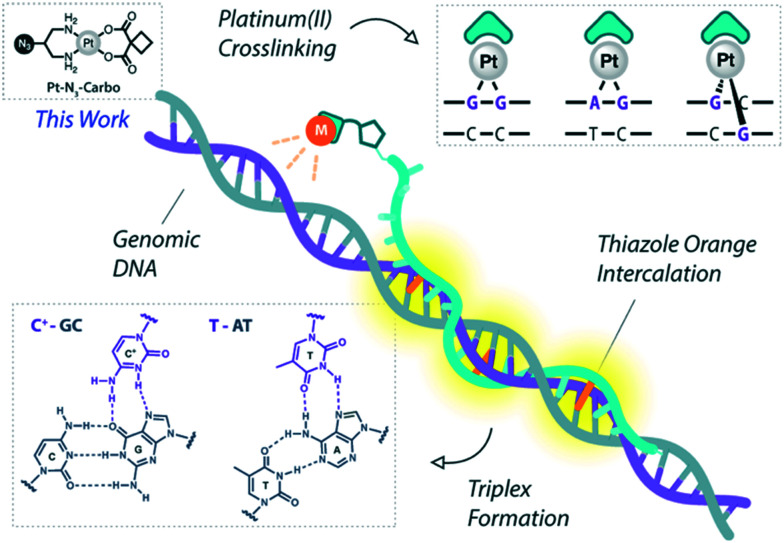
Pt(ii)-TFO hybridisation with genomic DNA target. Thiazole orange intercalation provides enhanced stabilisation. This work involves the use of Pt–N_3_-Carbo.

**Fig. 2:**
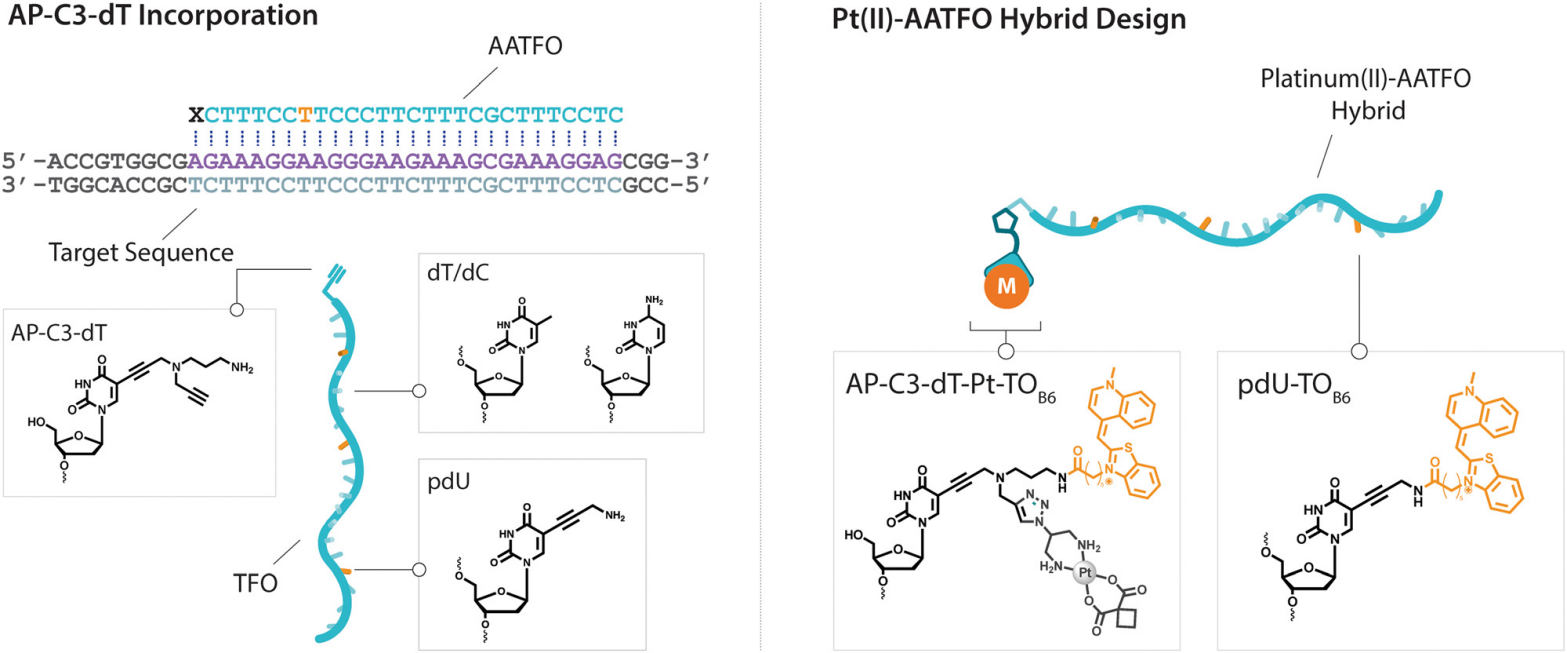
Left. TFO and target sequence design displaying AP-C3-dT incorporation. TFO sequence is composed of AP-C3-dT (**X**), dT/dC (**T**/**C**) and pdU nucleobases (**U**). Right. Scheme representing Pt(ii)-AATFO hybrid clicked and labelled with Pt–N_3_-Carbo and TO_B6_.

Platinum metallodrugs remain a core tenet of modern anticancer drug research.^1^ However, limitations of their use include systemic toxicity and poor target discrimination.^2^ In recent years, antigene technology^3^-specifically triplex-forming oligonucleotides (TFOs)^4–6^-has emerged as an ideal vector for directing traditional platinum agents.^7–10^ These modern strategies target extended genomic tracts while overcoming shortcomings in the targeting limitations of small molecule platinum drugs.^11^ Precise targeting is the key impact of this technology with platinum nucleic acid hybrids achieving effective gene-knockdown *in vitro*.^8^ We recently reported the generation of azide-modified *cis*-platinum(ii) complexes and showed how their copper(i)-catalysed azide–alkyne cycloaddition (CuAAC) ‘click chemistry’ attachment to TFOs yielded effective platinum(ii) nucleic acid hybrids (Fig. 1). These hybrids displayed targeting affinity combined with efficient triplex-mediated crosslinking.^9,12^ Herein, we report the development of enhanced combination probes that facilitate both the click chemistry modification of TFOs with an azide-functionalised carboplatin analogue (Pt–N_3_-Carbo), and the coupling of a thiazole orange (TO_B6_)^13–15^ fluorogenic intercalator. Click chemistry has recently been employed as an effective method for the direction and detection of azide-appended Pt(ii) metallodrugs.^16^ The use of a unique bifunctional thymine nucleobase (AP-C3-dT)^17^ enables incorporation of both the platinum agent and the TO_B6_ intercalator on a single nucleobase (Fig. 2). Thiazole orange enhances both the stabilisation and detection of DNA triplexes^15,18^ and is an effective adjuvant for probing DNA (triplex and G-quadruplex motifs)^19^ and RNA.^20^ Furthermore, applications of thiazole orange include its use as a surrogate base in PNA hybrids.^21^ Whilst previous iterations of click Pt(ii)-TFO hybrids displayed target destabilisation,^9^ in the current work, we demonstrate that introduction of the AP-C3-dT monomer and TO_B6_ significantly enhances the stability of Pt(ii)-TFO hybrids by mitigating the destabilising effects of platinum(ii) crosslinking while simultaneously serving as a reporter for triplex formation. This study investigates the incorporation of a platinum drug and a fluorogenic intercalating probe on a single nucleobase.

## Results and discussion

### Development of Pt(ii)-AATFO hybrids

The platinum agent *cis*-[Pt(2-azidopropane-1,3-diamine)(CBDCA)] (Pt–N_3_-Carbo) was synthesised as previously described.^9^ Briefly, starting from K_2_PtCl_4_, Pt–N_3_-Carbo was isolated using a three-step procedure with 2-azidopropane-1,3-diamine as the azide-modified ligand. Thiazole orange (TO_B6_) was generated as reported by Klimkowski *et al.*^15^ The benzothiazole derivative was selected instead of the quinoline derivative (TO_Q6_)^18,22^ as it is known to have a greater stabilising effect within DNA triplexes. TFOs were synthesised by solid-phase synthesis as described in experimental section 1.2. Nucleobase labelling experiments with TO_B6_ and the click chemistry conjugation of Pt–N_3_-Carbo were then performed (experimental sections 1.3 and 1.4). A series of three TFOs were generated to target a precise location of the green fluorescent protein (GFP) gene. We designed TFOs for enhanced triplex formation by: i) selecting a homopyrimidine sequence for parallel Hoogsteen binding with the duplex target; ii) minimising the number of consecutive C^+^-GC triplets in the DNA triplex to prevent destabilisation at lower pH; and iii) limiting the number of destabilising base inversions.^23^ The rationale for using a combination probe was twofold: firstly, labelling the AP-C3-dT nucleobase with TO_B6_ in conjunction with the platinum agent may enhance stability compared to the positioning of these separate modifications within the extended TFO sequence. Secondly, the bifunctional labelling should provide an anchoring effect toward the Pt–N_3_-Carbo moiety, thereby bringing the DNA reactive platinum(ii) drug more directly into contact with the nucleic acid interface. Without the use of AP-C3-dT, both of these enhancements would otherwise not be possible to achieve. In our probe design, additional thiazole orange units were incorporated to enhance stabilisation and crosslinking (Fig. 2). TFO constructs were then prepared through nucleic acid click chemistry by combining Pt–N_3_-Carbo and a series of alkyne–amine (AA)-TFOs (Table S1†). The TFOs are 28 nt sequences that include the AP-C3-dT monomer (X) at the 5′-terminus and up to two 5-(1-propargyl)-dU monomers, capable of bearing additional TO units, were introduced within the oligomer. The full list of TFOs and targets developed are shown in Table 1.

**Table 1:** Sequences used in this work. Navy bases (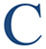, 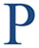 and 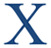) indicate a modification where 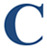 = (5)octadiynyl-deoxycytosine; 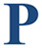 = (5)octadiynyl-deoxycytosine with Pt–N_3_-Carbo clicked; 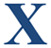 = AP-C3-dt coupled with Pt–N_3_-Carbo and TO_B6_. Red bases (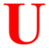) indicate pdU incorporation with TO_B6_ labelling. The light blue tract in the GFP target indicates the triplex recognition site

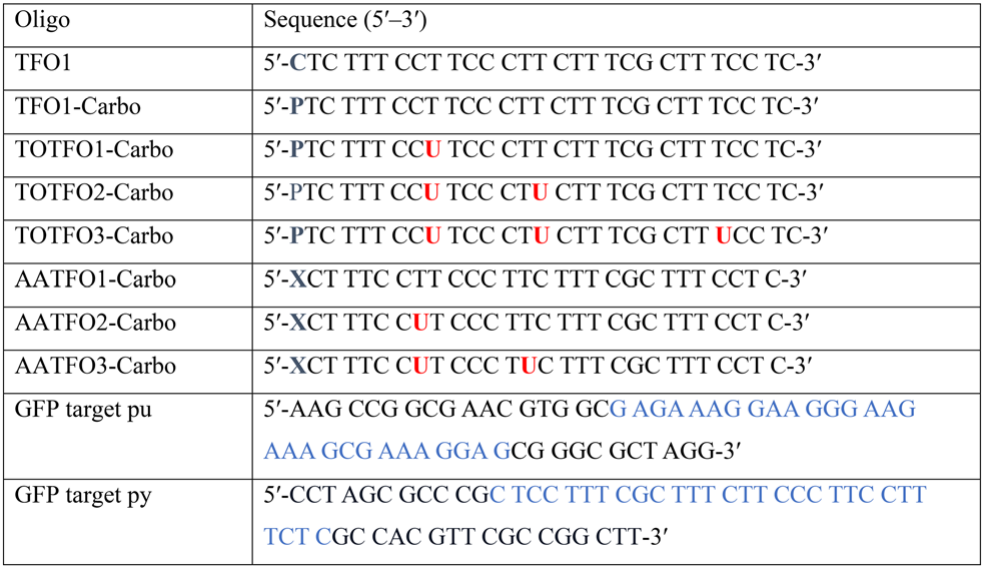

### Triplex-mediated crosslinking and stabilisation

**Fig. 3:**
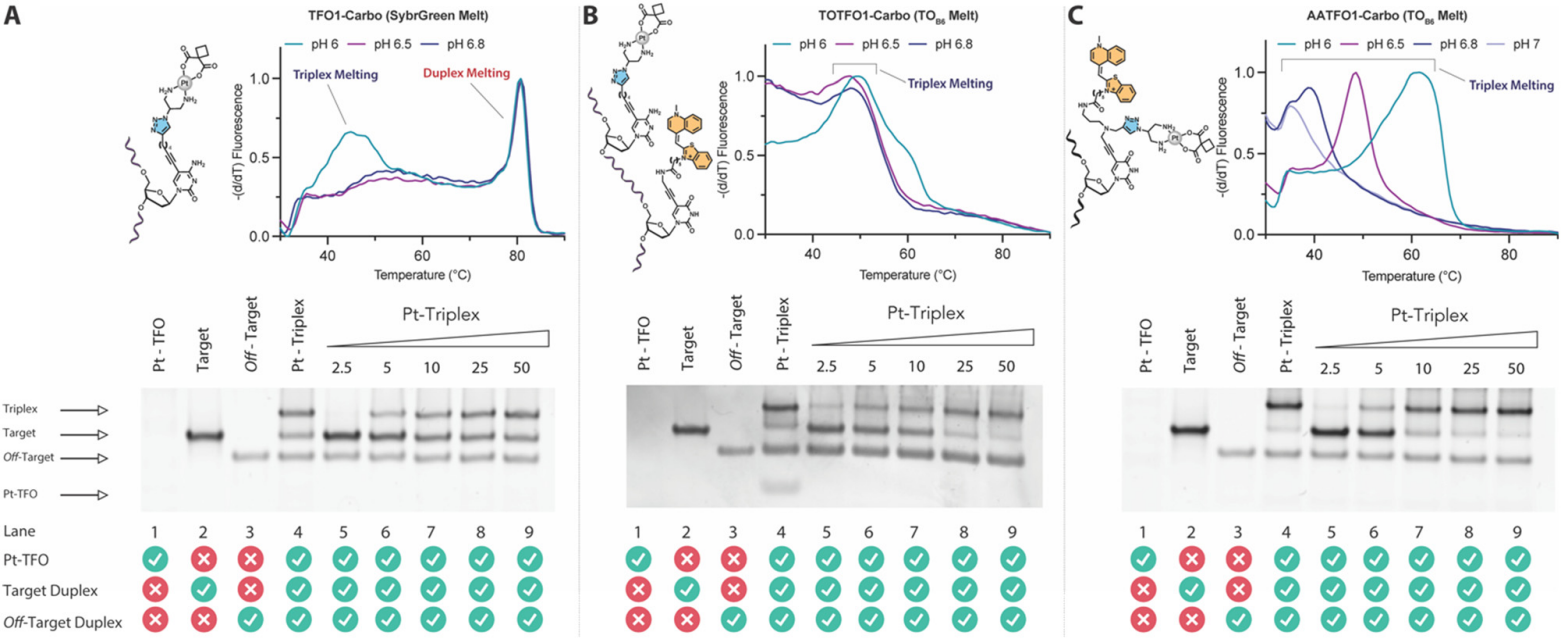
Fluorescence melting curve and PAGE analysis of triplex crosslinking for TFO1-Carbo, TOTFO1-Carbo, and AATFO1-Carbo. A. Fluorescence melting and PAGE for TFO1-Carbo with schematic of the platinum(ii)-clicked octadinyl-dC nucleobase. Triplex/duplex melting transitions are identified through SybrGreen addition. B. TOTFO1-Carbo with schematic displaying the platinum(ii)-clicked octadinyl-dC nucleobase and separate TO labelled propargyl-dU modification. C. AATFO1-Carbo with platinum(ii)-clicked and TO labelled AP-C3-dT nucleobase. Triplex melting transitions for TOTFO1- and AATFO1-Carbo are identified through intrinsic TO fluorescence. Lane 4 in all gels represents Pt(ii)-TFO hybrid loading of 50 eq. as a comparison for the gradient. Pt(ii)-TFO (lane 1) is generally not visible with SybrGold staining. Fluorescence melting samples were analysed in triplex melting buffer (10 mM phosphate 150 mM NaCl 2 mM MgCl_2_, pH 6.0–7.0, E1.5) and PAGE analysis for all TFOs was performed in TA triplex buffer (50 mM Tris-acetate, pH 6.0) at 70 V for 240 min. Full details of conditions are described in E1.6.

**Fig. 4:**
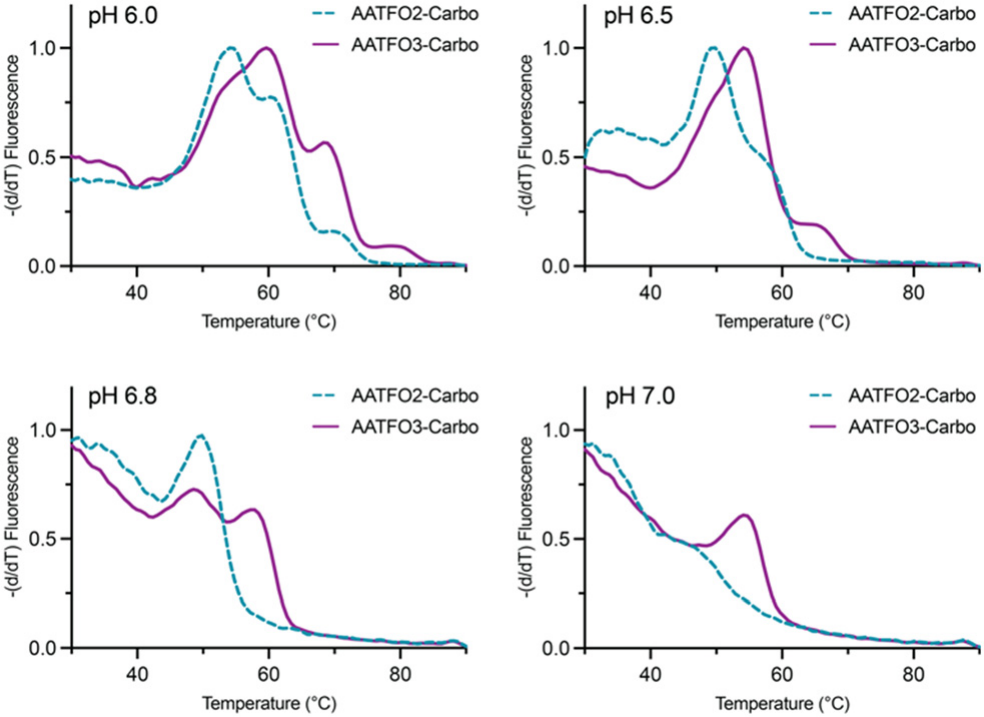
Fluorescence melting of AATFO2-Carbo and AATFO3-Carbo at pH 6.0, 6.5, 6.8, and 7.0. Samples were analysed in triplex melting buffer (E1.5).

TFO hybrids were used to target 40 and 57 base pair tracts of the GFP gene. Fluorescent melting experiments were performed in parallel with polyacrylamide gel electrophoresis (PAGE) to interrogate triplex formation between pH 6.0–7.0. TFO1, an alkyne-modified 29 mer TFO, and TFO1-Carbo—the same sequence but with a *cis*-[Pt(2-azidopropane-1,3-diamine)(CBDCA)] clicked to the 5′-terminal octadinyl-dC—displayed melting temperatures at pH 6.0 of 45.7 °C and 44.3 °C, respectively (Fig. 3A and Table S2†). The minor destabilisation incurred by TFO1-Carbo is consistent with our previous findings that *cis*-Pt(ii)-TFO hybrids induce crosslink-dependent destabilisation of the underlying duplex target which also affects triplex formation.^9^ Both TFO1 and TFO1-Carbo (Fig. 3A) failed to form stable triplexes at pH 6.5, 6.8, and 7.0. TOTFO1-Carbo, a 29 nt TFO with Pt-N3-Carbo clicked to the 5′-octadinyl-dC and with an internally labelled TOB6-pdU, demonstrated triplex formation with consistent melting temperatures identified at pH 6.0 and 6.5 (Fig. 3B and Table S2†). However, it was not possible to conclusively identify a DNA triplex at pH 6.8, while no formation was observed at pH 7.0 (Table S2†). In contrast, AATFO1-Carbo, a 28 nt hybrid where the Pt–N_3_-Carbo and TOB6 are situated on the same AP-C3-dT nucleobase, displayed triplex formation across the entire pH range. At pH 6.0, a melting temperature of 62.8 °C was observed (Fig. 3C). This melting temperature decreased with increasing pH and, at pH 7.0, the hybrid reported a near-physiological melting temperature of 35.4 °C (Table S2†). In comparison, AATFO2-Carbo, which contains an additional TO modification, and AATFO3-Carbo, which contains two additional TO units, showed improved triplex melting temperatures across the entire pH range (Fig. 4). The melting profiles for these TFOs are, however, more complex and display transitions consistent with the dissociation of multiple fluorophores from the crosslinked target. The major transitions associated with triplex melting events are presented in Table S2.† Significantly, at pH 7.0, AATFO2-Carbo and AATFO3-Carbo recorded melting temperatures of 48.7 °C and 54.1 °C, respectively, thereby demonstrating enhanced potential. With triplex formation established at various pH values, the platinum crosslinking capability of the series was examined across a range of concentrations. PAGE analysis of triplex crosslinking hybrids TFO1-Carbo, TOTFO1-Carbo, and AATFO1-Carbo was performed as described in experimental 1.6. Here, a 57 bp duplex target from the eGFP gene was investigated alongside a 40 bp off-target scrambled sequence (ESI† S1). Importantly, the off-target contained multiple GG and AG sites to encourage platinum(ii) crosslink formation, however, this sequence does not contain the triplex recognition element of the GFP target duplex. Concentration-dependent triplex formation was evident for all three hybrids (Fig. 3A–C, lanes 5–9) with a single higher order triplex crosslink band observed. Importantly, there was no ablation of the off-target duplex demonstrating that the platinum(ii) TFO was selective to its target. TFO1-Carbo, however, failed to generate complete triplex formation, and even at the highest concentrations of 50 equivalents (eq.) a mixture of target duplex and triplex exist (Fig. 3A, lane 9). TOTFO1-Carbo, on the other hand, demonstrated almost complete triplex crosslinking at between 10–25 eq. (Fig. 3B, lanes 7–8). It is clear, therefore, that at pH 6.0 the presence of intercalating TO provides significant stability and remediates some of the destabilising effects arising from platinum(ii) adduct formation. In comparison to TOTFO1-Carbo, AATFO1-Carbo demonstrated almost complete triplex formation between 5–10 eq. (Fig. 3C, lanes 6–7) which shows that incorporating both the platinum(ii) agent and the TO intercalator on the same nucleobase leads to enhanced targeting activity and enables parallel the monitoring of triplex formation without exogenous staining (Fig. S10A†). Finally, while AATFO2-Carbo displayed identical PAGE triplex formation properties to AATFO1-Carbo, AATFO3-Carbo consumed the target duplex more efficiently at the lowest tested concentration of 2.5 eq. (Fig. S8C†).

### Identification of crosslink formation

**Fig. 5:**
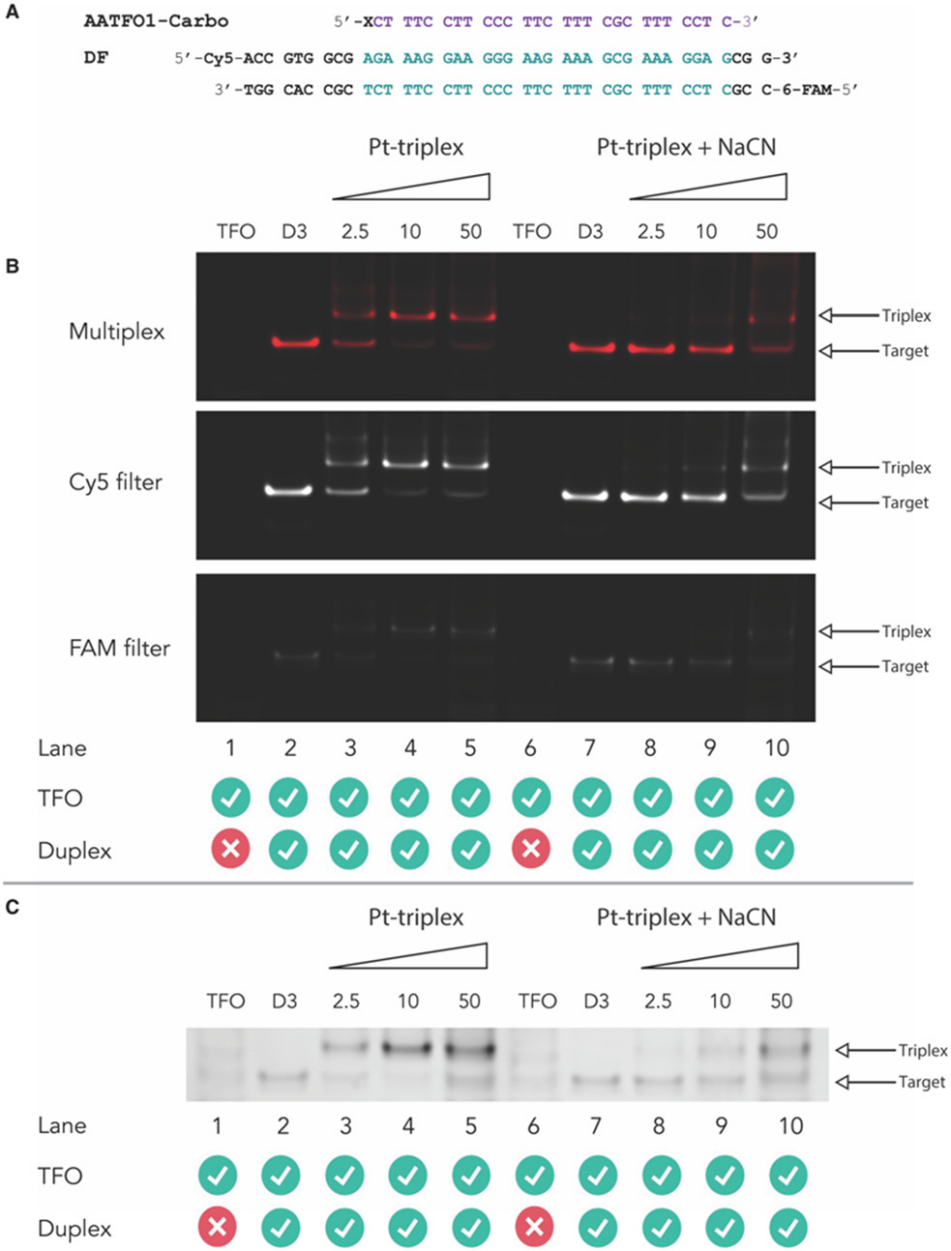
A. AA-TFO1-Carbo and fluorescently labelled target DF with triplex recognition site identified. B. Triplex-mediated crosslinking of the DF target sequences at 2.5, 10 and 50 eq. (lanes 3–5). Treatment of Pt(ii)-triplex system with 5000 eq. NaCN demonstrates reversal of the crosslinking formed by the hybrid (lanes 8–10). C. Pt(ii)-triplex system and NaCN reversal post SYBR gold staining.

Next, crosslinking mechanistic experiments were performed for the AATFOs using a sodium cyanide (NaCN) reversal assay. Here, a 40 bp duplex target containing the recognition site, DF (Fig. 5A), was treated with the AATFO-Carbo series (2.5, 10 and 50 eq.) (Fig. 5B, lanes 3–5) in the absence of NaCN to show crosslinking activity. The single crosslinked band produced by Pt(ii)-TFOs was also evident in the AATFO-Carbo experiments. Both AATFO2- and AATFO3-Carbo showed similar activity and underwent subsequent reversal upon NaCN treatment (Fig. S11†). Once treated with 5000 eq. of NaCN—the point at which triplex and native duplex are in equilibrium^9^—full reversal of AA-TFO-Carbo crosslinking was identified (Fig. 5B, lanes 8–10). Overall, while TOTFO1-Carbo is an effective probe, AATFO1-Carbo clearly outperforms this TFO. Although both of these sequences contain a single TO_B6_ modification, positioning TO_B6_ on the same nucleobase as the platinum(ii) drug augments the melting temperature by +11.9 °C. Furthermore, through PAGE analysis, the AATFO1-Carbo hybrid was found to consume its target duplex at significantly lower concentrations compared to TOTFO1-Carbo. Finally, there is a notable enhancement to the AATFO performance at higher pH when additional TO units are present with these probes demonstrating sequence-specific binding at neutral pH.

## Conclusions

In summary, *cis*-platinum(ii)-TFO hybrids containing the AP-C3-dT nucleobase represent a new design strategy in the development of click chemistry-based metallodrug hybrids. By incorporating a dual platinum(ii) drug and an intercalating thiazole orange fluorophore moiety, enhanced stability over other TFO designs—*e.g.* where the thiazole orange is dissociated from the platinum(ii) unit within the sequence—was observed. Indeed, the presence of TO appears essential to the design of Pt(ii)-TFOs given the performance of non-TO-modified constructs. Significantly, triplex-mediated platinum crosslinking was observed at much lower concentrations for the AATFOs, indicating the utility of the AP-C3-dT nucleobase. In agreement with previous work involving thiazole orange-modified TFOs,^18^ the incorporation of two intercalating units appears to be optimal for this type of probe design. Additionally, since nucleic acid click chemistry is modular in nature, there is flexibility to introduce alternative platinum crosslinking agents into this general scaffold. The employment of modified bases such as 5-methylcytosine,^24^ pseudoisocytosine,^25^ and 6-amino-5-nitropyridin-2-one^26^ (*Z*) may further increase the performance of these hybrids and allow for investigation at pH values above 7.

## Experimental

### 1. Materials and general information

**Fig. 6:**
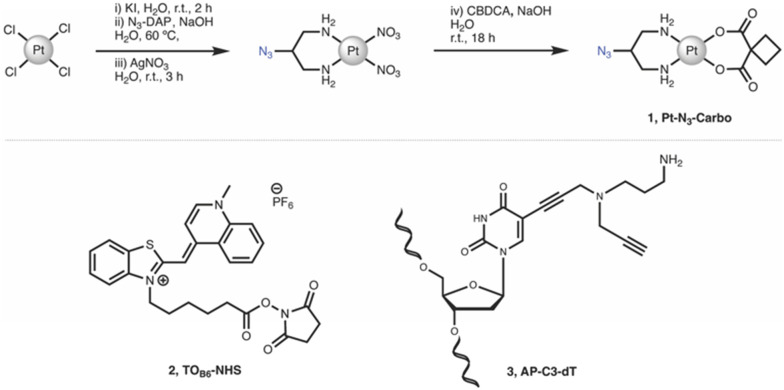
Synthetic route and structure for (1) Pt–N_3_-Carbo. Structures for (2) TO_B6_-NHS and (3) AP-C3-dT.

Chemicals, reagents and HPLC grade solvents were sourced from Sigma-Aldrich (Ireland) Ltd., unless otherwise stated, and were used without any further purification. Dichloromethane (DCM) was distilled from calcium hydride and stored under argon. All other solvents were used as supplied. ^1^H-NMR, ^13^C-NMR and ^195^Pt-NMR spectra were obtained on a Bruker AC 600 MHz NMR spectrometer and processed using MNova (MestreLab). FT-IR spectra were obtained from neat solids on a Perkin Elmer Spectrum Two spectrometer. Melting points were obtained on a Stanford Research Systems MPA100 Optimelt apparatus. ESI-MS analysis was performed on a MaXis HD ESI-QTOF mass spectrometer (Bruker Daltonik GmbH) with data processing performed using Compass Data Analysis software (v4.3, Bruker Daltonik GmbH). PCR, triplex and duplex annealing were performed using a Mastercycler nexus (Eppendorf). Fluorescent Thermal Melting Analysis was performed on a Roche LightCycler 480 II using SYBR green I master mix (Roche). PAGE gels were cast using a SureCast gel casting system (Invitrogen) and run in an Invitrogen mini gel tank system. Gels were run using a Bio-Rad basic Power Pac system and imaged using a Syngene G:Box 9 mini gel documentation system. **Important note**: organic azides are hazardous materials and are known to be heat- and shock- sensitive. Explosive decomposition can potentially occur with very little energy input. To ensure safe manipulation and non-explosiveness for organic azides, the rule is that the number of nitrogen atoms must not exceed that of carbon, where (*N*_C_ + *N*_O_)/*N*_N_ ≥ 3 (*N* = number of atoms).^27^ Each azide should be individually evaluated. 2-Azidopropane-1,3-diamine (N_3_-DAP) (Fig. 6) is a potentially hazardous material, however all relevant safety precautions were undertaken while working with the material.

#### 1.1 Synthesis

##### *cis*-[Pt(2-Azidopropane-1,3-diamine)(CBDCA)] (1, Pt-N_3_-Carbo)

Pt–N_3_-Carbo was synthesised as previously reported.^9^ In brief, *cis*-[Pt(2-azidopropane-1,3-diamine)I_2_] intermediate was suspended in DI H_2_O and an aqueous solution of AgNO_3_ was added dropwise with stirring. The solution was stirred for 3 h before filtration to remove AgI. To the filtrate was added an aqueous solution of cyclobutane-1,1-dicarboxylic acid (CBCDA) and NaOH. The solution was stirred for approx. 18 h in the dark and filtered prior to the removal of solvent to yield a crude grey residue. A small volume of DI H_2_O was added to the residue and cooled at 5 °C before vacuum filtration, washed with H_2_O, MeOH and Et_2_O and finally dried to obtain an off-white solid as product. ^1^H NMR (600 MHz, DMSO-*d*_6_) *δ* 5.45 (s, 2H), 5.28 (s, 2H), 3.91 (s, 1H), 2.65 (quintuplet, 4H), 2.53 (m, 2H), 2.48 (m, 2H), 1.64 (quintuplet, 2H). ^13^C NMR (151 MHz, DMSO-*d*_6_): 177.89, 59.16, 56.06, 45.86, 31.04, 30.71, 15.45. ^195^Pt NMR (129 MHz, DMSO-*d*_6_) *δ* −1942. IR (ATR, cm^−1^): 3217, 3092, 2938, 2120, 1622, 1576, 1342, 1268, 1106, 1024, 900, 768. ESI-MS: *m*/*z*. 453.1 [M + H]^+^, 475.1 [M + Na]^+^. ESI-MS calc. for C_9_H_15_N_5_O_4_^195^Pt^+^ [M + H]^+^: 453.0850. Found: 453.0846.

##### *N*-(5-Carboxypentyl)-2-[(1,4-dihydro-1-methylquinolin-4-ylidene)methyl]benzothiazole-3-ium *N*-hydroxysuccinimide ester hexafluorophosphate (2, TO_B6_-NHS)

TO_B6_-NHS was synthesised and characterised according to the literature procedure reported by Klimkowski *et al.*^15^

##### 5-Propargylamino(*N*-propargyl-*N*-propyl-2,2,2-trifluoroacetamide)-5′-*O*-(4,4′-dimethoxy-tritryl)-2′-deoxythymidine diisopropylamino cyanoethyl phosphoramidite (3, AP-C3-dt)

AP-C3-dt was synthesised as previously reported by Bai *et al.*^17^

#### 1.2 Oligonucleotide synthesis

Solid supports, standard DNA phosphoramidites and all other reagents used in the synthesis were purchased from Sigma Aldrich. Modified phosphoramidites were purchased from Glen Research. Oligonucleotides (ODNs) were synthesised on a K&A Laborgeräte H-8-SE LNA, DNA/RNA synthesiser using the standard 1.0 μmol phosphoramidite cycle. Coupling efficiencies were monitored by the trityl cation conductivity monitoring facility and was >98% for all oligonucleotides. Standard monomers (A, G, C, and T) were coupled for 35 s and non-standard monomers were coupled for 360 s. ODNs were deprotected and cleaved from the solid support using a concentrated ammonia solution for 1 h at room temperature, followed by heating in sealed vials for 5 h at 55 °C. ODNs were purified using reverse-phase HPLC on a Gilson HPLC system using a Luna 10 μm C8 100 Å 250 × 10 mm column. For standard alkyne-modified ODNs, the gradient was 10–45% buffer B over 20 min with a flow rate of 4 mL min^−1^ (buffer A: 0.1 M triethylammonium acetate (TEAA), buffer B: 0.1 M TEAA with 50% MeCN). Fraction volumes were reduced by rotary evaporation prior to redissolution in water and desalted using NAP-10 gel filtration columns purchased from GE Healthcare. All ODNs were characterised by negative-mode HPLC-mass spectrometry using a Waters Xevo G2-XS QT mass spectrometer with an Acquity UPLC system equipped with an Acquity UPLC oligonucleotides BEH C18 column (particle size: 1.7 μm; pore size 130 Å; column dimensions: 2.1 × 50 mm). Data was deconvoluted and analysed using Waters Mass Lynx software. Oligonucleotide synthesis with modified nucleobases was performed as described above with some minor deviations. Standard monomers (A, G, C, and T) were coupled for 35 s and non-standard monomers were coupled for 360 s. ODNs with incorporated pdU additions and those that were labelled with thiazole orange (TO_B6_-NHS) or fluorescein/cyanine-5 dyes were purified with ammonium acetate (NH_4_OAc) with a gradient of 15–45% buffer B over 25 min, flow rate of 4 mL min^−1^ (buffer A: 0.1 M NH_4_OAc, buffer B: 0.1 M NH_4_OAc 50% MeCN). The 40 bp off-target sequence (puC19, NEB; forward: 5′-TGACTCCCCGTCGTGTAGAT-3′ and reverse: 5′-AGCCCTCCCGTATCGTAGTT-3′) was generated by PCR (MyTaq Red DNA polymerase, Bioline) and purified into 6 μL nuclease-free water using membrane spin columns (Monarch PCR & DNA clean-up kit, NEB).

#### 1.3 Thiazole orange (TO) oligonucleotide labelling

Oligonucleotide labelling was performed as previously described,^9^ with minor changes. In a total volume of approx. 200 μL, 2000 nmol (10 eq.) of TO_B6_ NHS ester in DMF was added to 200 nmol of oligonucleotide dissolved in carbonate buffer (NaHCO_3_/Na_2_CO_3_, 0.5 M, pH 8.74). The solution was shaken at 25 °C for 3 h prior to desalting by NAP gel filtration column and purified by reverse-phase HPLC utilising a C8 column with a gradient of 0.1 M NH_4_OAc + MeCN (50%) (buffer B) in 0.1 M NH_4_OAc (buffer A). ESI-MS analysis, as previously described was used to determine the purity of the labelled oligonucleotide. For ODNs that contained two or three labelling sites, 20 or 40 eq. of TO_B6_ NHS ester was used respectively. For AA-TFOs that were clicked to *cis*-[Pt(2-azidopropane-1,3-diamine)(CBDCA)], no purification of the TO labelled oligo was performed. Purification was only performed after click conjugation to ensure optimum yield.

#### 1.4 *cis*-Platinum(ii)-TFO hybrid generation using click chemistry

A previously described click chemistry hybridisation protocol was used to obtain *cis*-Pt(ii)-TFO and *cis*-Pt(ii)-TOTFO.^9^ In brief, in a total volume of 500 μL, 1 : 1 H_2_O : DMSO, 10 eq. of Pt(ii)–azide complex was added to 1 eq. of alkyne-modified TFO, degassed with argon and 10 eq. of Cu-TBTA complex was added. Finally, 20 eq. Na-l-Ascorbate was added and the solution was degassed prior to stirring at 25 °C for 3–6 h. For the *cis*-platinum(ii)-AATFO hybrids, the solutions were stirred at 25 °C for 6–8 h. The solutions were desalted by elution through NAP gel filtration column and purified and characterised following the oligonucleotide purification protocol described above.

#### 1.5 Fluorescent thermal melting analysis

Target duplex samples were dissolved in appropriate triplex buffer (10 mM phosphate, 150 mM NaCl, 2 mM MgCl_2_ pH 6–7) and annealed by heating to 95 °C and slowly cooling (1 °C min^−1^) to 4 °C prior to addition of TFO/Pt(ii)-TFO. Sample solutions were incubated at 37 °C for 48 h. Samples were analysed using a previously described method.^9^ SYBR green master mix I (1 μL, Roche) was added to each sample solution and the melting profile for the triplex crosslink was analysed on a LightCycler 480 II (Roche). For samples containing TO-modified oligonucleotides, no SYBR green master mix I was added. Samples were heated to 99 °C at a rate of 0.1 °C s^−1^ with ten fluorescence measurements recorded per °C. A plot of sample fluorescence *versus* temperature is obtained, normalised and the first negative derivative of the sample was calculated. Samples were analysed in triplicate and *T*_M_ was calculated as an average of the first negative derivative of the melting curve. Melting curves were analysed and graphed using GraphPad Prism 9 software.

#### 1.6 Triplex-mediated crosslink formation

Target (57 bp, 1 pmol) and off-target (40 bp, 1 pmol) duplexes were treated with increasing concentrations of Pt(ii)-TFO hybrid (2.5–50 eq.) in triplex buffer (10 mM phosphate, 150 mM NaCl, 2 mM MgCl_2_ pH 6–7) and incubated at 37 °C for 48 h. Orange gel loading dye (1 μL, 6X, NEB) was added and the sample solution was loaded onto a 20% PAGE gel (50 mM Tris acetate, 150 mM NaCl, 2 mM MgCl_2_, pH 6–7). Electrophoresis was performed at 70 V for 240 min in triplex running buffer (50 mM Tris acetate, pH 6–7). Polyacrylamide gels with TO-containing sequences were visualised and imaged on a Syngene G:Box Mini 9 gel documentation system. Subsequently, TO-containing sequences were then post-stained with SYBR Gold and visualised similarly. Gels with non-TO-containing sequences were post-stained with SYBR Gold and visualised as before.

#### 1.7 Crosslink reversal by sodium cyanide

The protocol for crosslink reversal was used as previously performed. In brief, fluorescently-labelled duplex target (**DF**, 40 bp, 1 pmol) was treated with Pt(ii)-AATFO (50 eq.). Samples were incubated at 37 °C for 48 h prior to addition of 5000 eq. of NaCN solution. Combined samples were incubated at r.t. for 18 h and quenched with 30% glycerol solution. PAGE electrophoresis was performed at 70 V for 240 min in triplex running buffer (50 mM Tris acetate, pH 6). Gel visualisation was performed on a Syngene G:Box Mini 9 gel documentation system with integral Cy5 and 6-FAM filters.

## Conflicts of interest

There are no conflicts to declare.

## Supplementary Material

MD-015-D3MD00548H-s001

## References

[cit1] Alassadi S., Pisani M. J., Wheate N. J. (2022). Dalton Trans..

[cit2] Rottenberg S., Disler C., Perego P. (2021). Nat. Rev. Cancer.

[cit3] Crooke S. T., Baker B. F., Crooke R. M., Liang X.-H. (2021). Nat. Rev. Drug Discovery.

[cit4] Vasquez K. M., Narayanan L., Glazer P. M. (2000). Science.

[cit5] Lohani N., Rajeswari M. R. (2020). Anti-Cancer Agents Med. Chem..

[cit6] McGorman B., Fantoni N. Z., O'Carroll S., Ziemele A., El-Sagheer A. H., Brown T., Kellett A. (2022). Nucleic Acids Res..

[cit7] Graham M. K., Miller P. S. (2012). J. Biol. Inorg. Chem..

[cit8] Graham M. K., Brown T. R., Miller P. S. (2015). Biochemistry.

[cit9] Hennessy J., McGorman B., Molphy Z., Farrell N. P., Singleton D., Brown T., Kellett A. (2022). Angew. Chem..

[cit10] Cunningham R. M., Hickey A. M., Wilson J. W., Plakos K. J. I., DeRose V. J. (2018). J. Inorg. Biochem..

[cit11] Guha R., Defayay D., Hepp A., Muller J. (2021). ChemPlusChem.

[cit12] Kellett (DCU) A. and Hennessy (DCU) J., WO2023046875, 2023

[cit13] Bethge L., Singh I., Seitz O. (2010). Org. Biomol. Chem..

[cit14] Hövelmann F., Seitz O. (2016). Acc. Chem. Res..

[cit15] Klimkowski P., De Ornellas S., Singleton D., El-Sagheer A. H., Brown T. (2019). Org. Biomol. Chem..

[cit16] Wirth R., White J. D., Moghaddam A. D., Ginzburg A. L., Zakharov L. N., Haley M. M., DeRose V. J. (2015). J. Am. Chem. Soc..

[cit17] Bai C. S., Klimkowski P., Jin C., Kuchlyan J., El-Sagheer A. H., Brown T. (2022). Org. Biomol. Chem..

[cit18] Walsh S., El-Sagheer A. H., Brown T. (2018). Chem. Sci..

[cit19] Lubitz I., Zikich D., Kotlyar A. (2010). Biochemistry.

[cit20] Tonelli A., Tedeschi T., Germini A., Sforza S., Corradini R., Medici M. C., Chezzi C., Marchelli R. (2011). Mol. BioSyst..

[cit21] Sato T., Sato Y., Nishizawa S. (2016). J. Am. Chem. Soc..

[cit22] Ikeda S., Yanagisawa H., Yuki M., Okamoto A. (2013). Artif. DNA PNA XNA.

[cit23] Rusling D. A. (2005). Nucleic Acids Res..

[cit24] Lee J. S., Woodsworth M. L., Latimer L. J., Morgan A. R. (1984). Nucleic Acids Res..

[cit25] Li H., Franzini R. M., Bruner C., Kool E. T. (2010). ChemBioChem.

[cit26] Rusling D. A. (2021). Nucleic Acids Res..

[cit27] Bräse S., Gil C., Knepper K., Zimmermann V. (2005). Angew. Chem., Int. Ed..

