# Supplementary material for: Thiazole orange-carboplatin triplex-forming oligonucleotide (TFO) combination probes enhance targeted DNA crosslinking
Source: RSC Med Chem. 2023 Oct 31;15(2):485–91. doi: 10.1039/d3md00548h (PMC10880910; doi:10.1039/d3md00548h)
Supplement: MD-015-D3MD00548H-s001 [file MD-015-D3MD00548H-s001.pdf]

Electronic Supplementary Material (ESI) for RSC Medicinal Chemistry.
This journal is © The Royal Society of Chemistry 2023

Supporting Information accompanying the manuscript

Thiazole Orange-Carboplatin Triplex-Forming Oligonucleotide (TFO) Combination Probes Enhance Targeted DNA Crosslinking

Joseph Hennessy,[a] Piotr Klimkowski,[b] Daniel Singleton,[c] Alex Gibney,[a] Malou Coche,[a] Nicholas P. Farrell,[d] Afaf H. El-Sagheer,[b,e,f] Tom Brown[b,c] and Andrew Kellett.[a]*

[a.] SSPC, the Science Foundation Ireland Research Centre for Pharmaceuticals, School of Chemical Sciences, Dublin City University, Glasnevin, Dublin 9, Ireland, Email: Andrew.kellett@dcu.ie

[b.] Chemistry Research Laboratory, University of Oxford, 12 Mansfield Road, Oxford OX1 3TA, United Kingdom.

[c.] ATDBio, Ltd., School of Chemistry, University of Southampton, Southampton, SO17 1BJ, United Kingdom.

[d.] Department of Chemistry, Virginia Commonwealth University, Richmond, VA 23284-2006, United States.

[e.]School of Chemistry, University of Southampton, Southampton, SO17 1BJ, United Kingdom.

[f.] Department of Science and Mathematics, Suez University, Faculty of Petroleum and Mining, Engineering, Suez 43721, Egypt.

List of Contents:

S1: Mass Spectroscopy Analysis of Oligonucleotides

S2: Fluorescent Thermal Melting Analysis of Pt(II)-TFO Hybrids

S3: Pt(II)-TFO Hybrid Click Chemistry Yields

S4: Pt(II)-TFO Hybrid PAGE

S5: Pt(II)-TFO Hybrid pH Comparison

S6: Fluorescent Triplex Formation

S7: NaCN Crosslink Reversal PAGE

S1: Mass Spectroscopy Analysis of Oligonucleotides

Table S1. Oligonucleotides, Pt(II)-TFO Hybrids and Duplex Targets. **AP-C3-dT** = 5-propargylamino(N-propargyl-N-propyl-2,2,2-trifluoroacetamide)-5'-*O*-(4,4'-dimethoxy-tritryl)-2'-deoxythymidine diisopropylamino cyanoethyl phosphoramidite; **pdU-TO** = 5-(1-propargylamino)-deoxyuridine with TO_{B6} coupled; **Pt-N$_3$-Carbo** = *cis*-[Pt(2-azidopropane-1,3-diamine)(CBDCA)]; **57 tgt** = 57 bp GFP duplex target; **DF tgt** = Duplex-Fluorophore purine target sequence; **DF comp** = Duplex-Fluorophore pyrimidine complimentary sequence.

Oligo	Sequence	Modification (X)(C)(U)	Calc. Mass	Obs. Mass
TFO1	5'-CTC TTT CCT TCC CTT CTT TCG CTT TCC TC-3'	5'-dC	8694.1	8694.3
TFO1-Carbo	5'-CTC TTT CCT TCC CTT CTT TCG CTT TCC TC-3'	5'-dC-N$_3$-Pt(II)-Carbo	9146	9146.1
TOTFO1-Carbo	5'-CTC TTT CCU TCC CTT CTT TCG CTT TCC TC-3'	5'-dC-N$_3$-Pt(II)-Carbo, pdU-TO (x1)	9572.3	9571.7
TOTFO2-Carbo	5'-CTC TTT CCU TCC CTU CTT TCG CTT TCC TC-3'	5'-dC-N$_3$-Pt(II)-Carbo, pdU-TO (x2)	9998.2	9997.4
TOTFO3-Carbo	5'-CTC TTT CCU TCC CTU CTT TCG CTT UCC TC-3'	5'-dC-N$_3$-Pt(II)-Carbo, pdU-TO (x3)	10424.3	10422.8
AATFO1-Carbo	5'-XCT TTC CTT CCC TTC TTT CGC TTT CCT C-3'	5'-AP-C3-dT-TO, 5'-AP-C3-dT-N$_3$-Pt(II)-Carbo	9274.0	9273.9
AATFO2-Carbo	5'-XCT TTC CUT CCC TTC TTT CGC TTT CCT C-3'	5'-AP-C3-dT-TO, 5'-AP-C3-dT-N$_3$-Pt(II)-Carbo, pdU-TO (x1)	9700.2	9699.1
AATFO3-Carbo	5'-XCT TTC CUT CCC TUC TTT CGC TTT CCT C-3'	5'-AP-C3-dT-TO, 5'-AP-C3-dT-N$_3$-Pt(II)-Carbo, pdU-TO (x2)	10126.0	10124.6
57 bp tgt.	5'-AAG CCG GCG AAC GTG GCG AGA AAG GAA GGG AAG AAA GCG AAA GGA GCG GGC GCT AGG-3'	n/a	17972.5	17972.8
57 bp comp.	3'-TTC GGC CGC TTG CAC CGC TCT TTC CTT CCC TTC TTT CGC TTT CCT CGC CCG CGA TCC-5'	n/a	17129.9	17129.7
DF tgt.	5'-Cy5- ACC GTG GCG AGA AAG GAA GGG AAG AAA GCG AAA GGA GCG G -3'	5'-Cyanine 5 label	13157.9	13156.9
DF comp.	3'- TGG CAC CGC TCT TTC CTT CCC TTC TTT CGC TTT CCT CGC C -6FAM-5'	5'-6-FAM label	12507.2	12505.8
40 bp off tgt.	5'- TGA CTC CCC GTC GTG TAG ATA ACT ACG ATA CGG GAG GGC T -3'	n/a	12336.9	n/a
40 bp off comp.	3'- ACT GAG GGG CAG CAC ATC TAT TGA TGC TAT GCC CTC CCG A -5'	n/a	12256.9	n/a

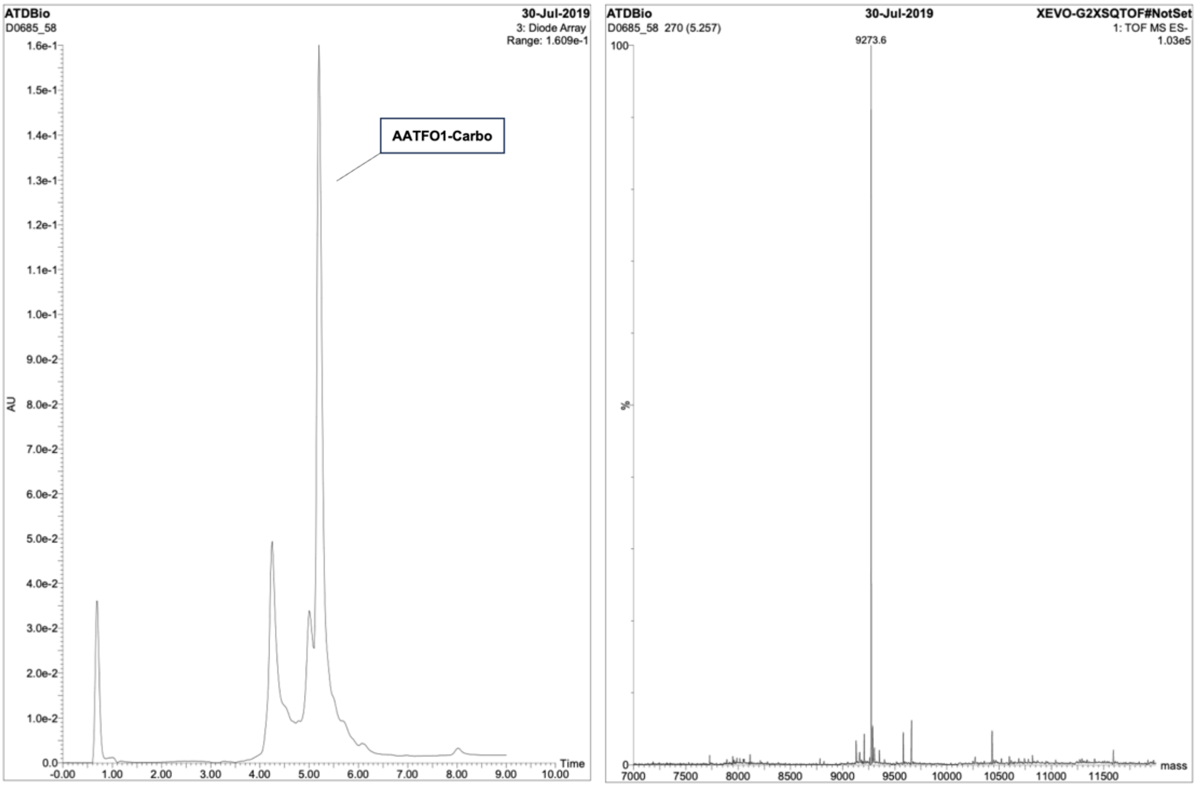

Figure S1. HPLC (**left**, x-axis = time (min) and y-axis = UV absorbance at 260 nm) and ESI-MS (**right**) spectra for **AATFO1-Carbo**.

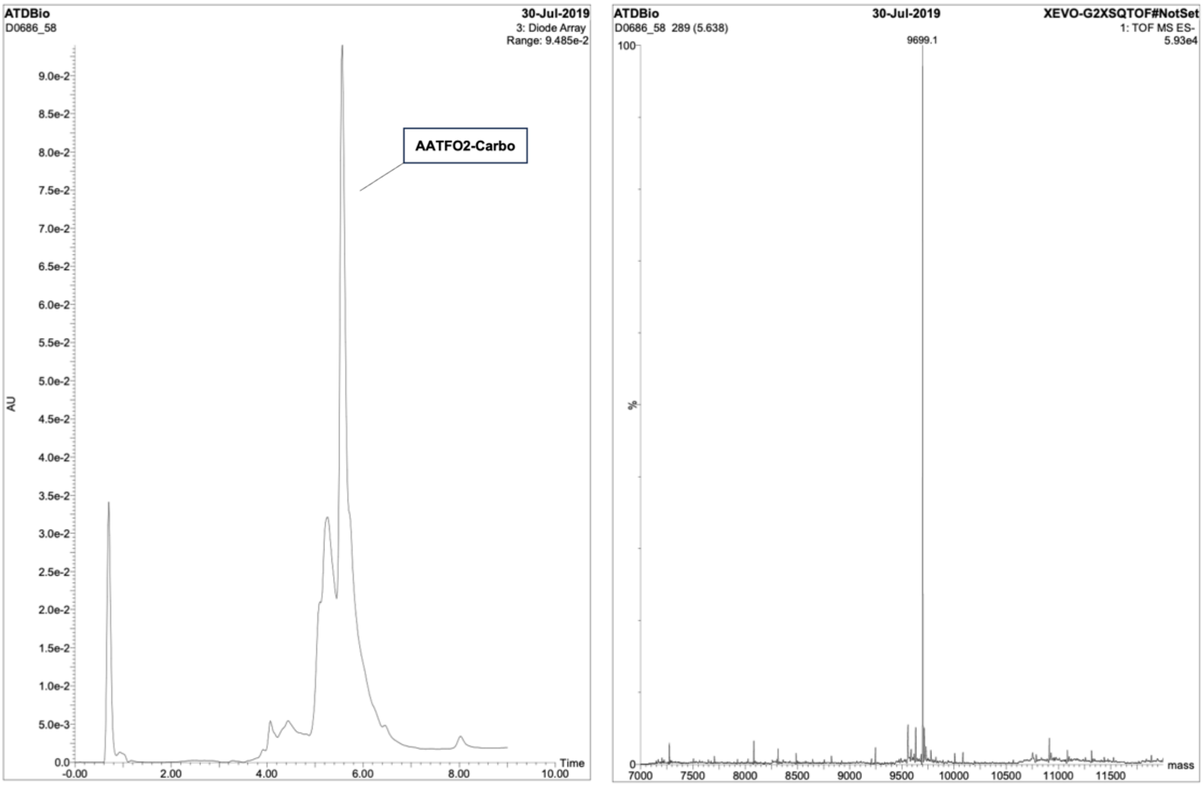

Figure S2. HPLC (**left**, x-axis = time (min) and y-axis = UV absorbance at 260 nm) and ESI-MS (**right**) spectra for **AATFO2-Carbo**.

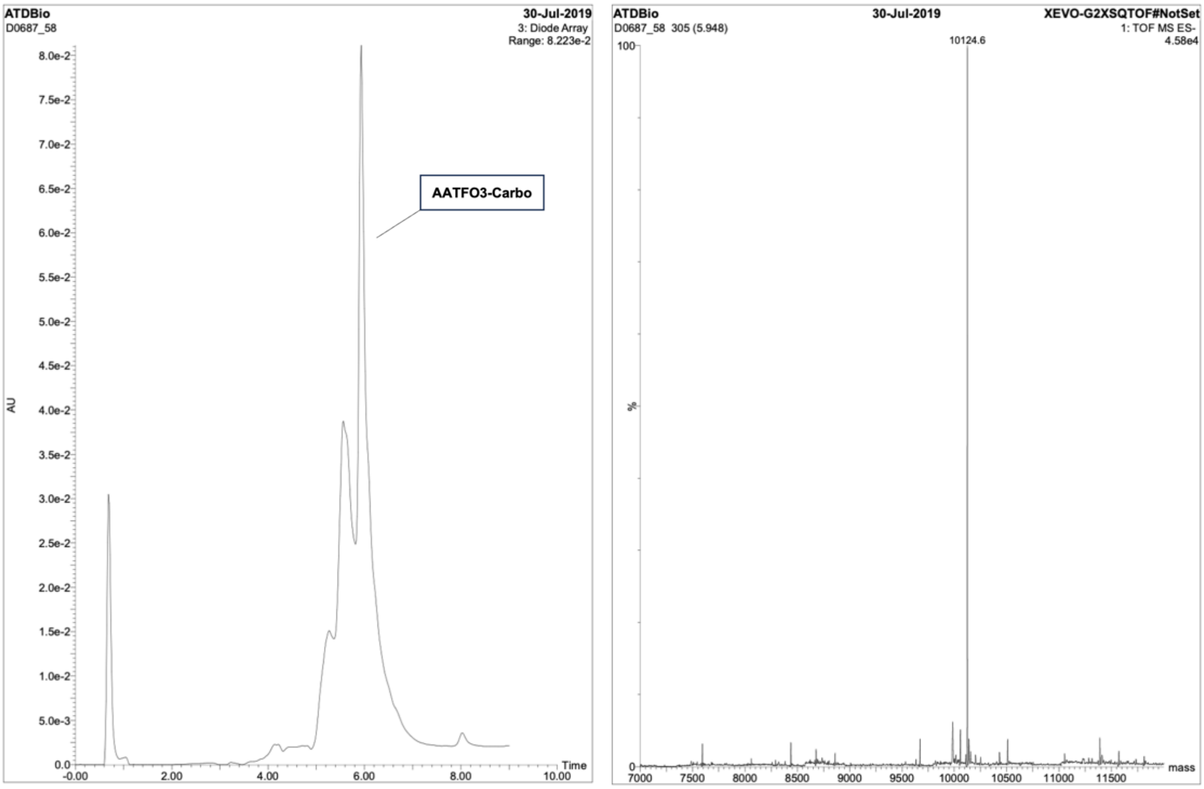

Figure S3. HPLC (**left**, x-axis = time (min) and y-axis = UV absorbance at 260 nm) and ESI-MS (**right**) spectra for **AATFO3-Carbo**.

S2. Fluorescent Thermal Melting Analysis of Pt(II)-TFO Hybrids

Table S2. T_M values were recorded in 10 mM phosphate (PO_4^{3-}), 150 mM NaCl, 2 mM $MgCl_2$, pH 6-7 buffer. Samples were incubated at 37 ºC for 48 h prior to melting analysis. T_M temperatures were calculated by the first negative derivative of the normalised fluorescence melting curve and graphed using GraphPad Prism 9.0. Final T_M values are an average of 3 melting samples. ΔT_M values for Pt(II)-TFOs are relative to the unclicked alkyne TFO precursor. **5'-dC** = 5'-(5)octadinyl-deoxycytidine; **pdU-TO** = 5-(1-propargylamino)-deoxyuridine with TO_{B6} coupled; **Pt-N$_3$-Carbo** = *cis*-[Pt(2-azidopropane-1,3-diamine)(CBDCA)]; **n.t.o.** = no triplex observed.

Triplex	Modification	pH 6.0		pH 6.5		pH 6.8		pH 7.0	
		T_M (°C)	ΔT_M	T_M (°C)	ΔT_M	T_M (°C)	ΔT_M	T_M (°C)	ΔT_M
**TFO1**	5'-dC	45.7 ± 0.5	-	n.t.o	-	n.t.o	-	n.t.o	-
**TFO1-Carbo**	5'-dC-N$_3$-Pt(II)-Carbo	44.3 ± 0.9	-1.4	n.t.o	-	n.t.o	-	n.t.o	-
**TOTFO1-Carbo**	5'-dC-N$_3$-Pt(II)-Carbo pdU-TO (x1)	50.9 ± 0.7	+ 5.2	49.6 ± 0.6	-	n.t.o	-	n.t.o	-
**TOTFO2-Carbo**	5'-dC-N$_3$-Pt(II)-Carbo pdU-TO (x2)	49.9 ± 0.4	+ 4.2	45.9 ± 0.8	-	n.t.o	-	n.t.o	-
**TOTFO3-Carbo**	5'-dC-N$_3$-Pt(II)-Carbo pdU-TO (x3)	48.5 ± 1.3	+ 2.8	45.8 ± 0.6	-	n.t.o	-	n.t.o	-
**AATFO1-Carbo**	5'-AP-C3-dT-TO, 5'-AP-C3-dT-N$_3$-Pt(II)-Carbo	62.8 ± 0.3	+ 17.1	49.3 ± 0.8	-	39.1 ± 0.2	-	35.4 ± 0.2	-
**AATFO2-Carbo**	5'-AP-C3-dT-TO, 5'-AP-C3-dT-N$_3$-Pt(II)-Carbo, pdU-TO (x1)	54.8 ± 1.1	+ 9.1	48.6 ± 0.2	-	50.1 ± 0.1	-	48.7 ± 0.4	-
**AATFO3-Carbo**	5'-AP-C3-dT-TO, 5'-AP-C3-dT-N$_3$-Pt(II)-Carbo, pdU-TO (x2)	60.3 ± 0.2	+ 14.6	54.50 ± 1.3	-	47.9 ± 0.3	-	54.1 ± 0.9	-
**Duplex**	**Sequence**	pH 6.0		pH 6.5		pH 6.8		pH 7.0	
**57 target**	57 bp	80.3 ± 0.1		80.2 ± 0.1		80.4 ± 0.1		80.4 ± 0.1	

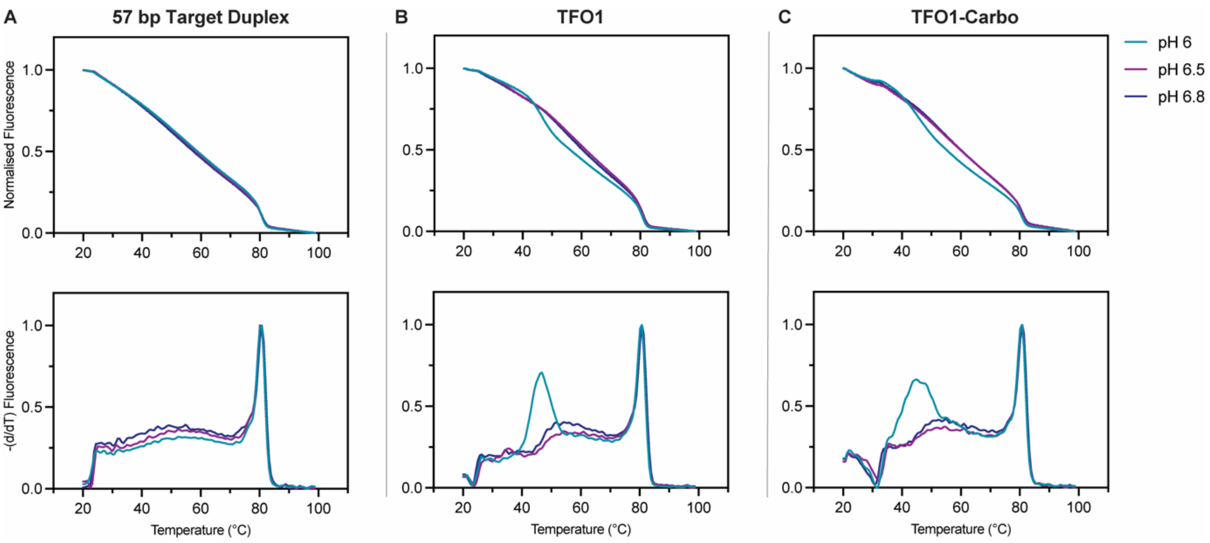

Figure S4. (**Top**) Normalised fluorescence melting curve plot and (**Bottom**) first negative derivative of fluorescence melting for (**A**) Duplex, (**B**) TFO1 and (**C**) TFO1-Carbo.

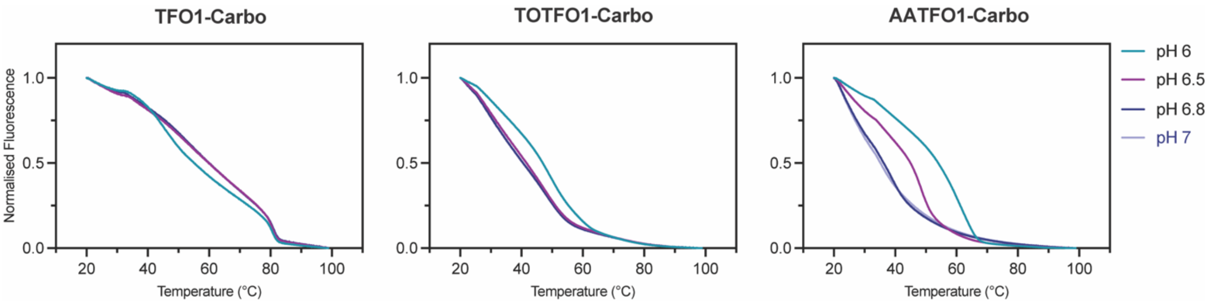

Figure S5. Normalised fluorescence melting curve plots for TFO1-Carbo, TOTFO1-Carbo and AATFO1-Carbo. Correspond to negative derivative melting plots for Figure 2.

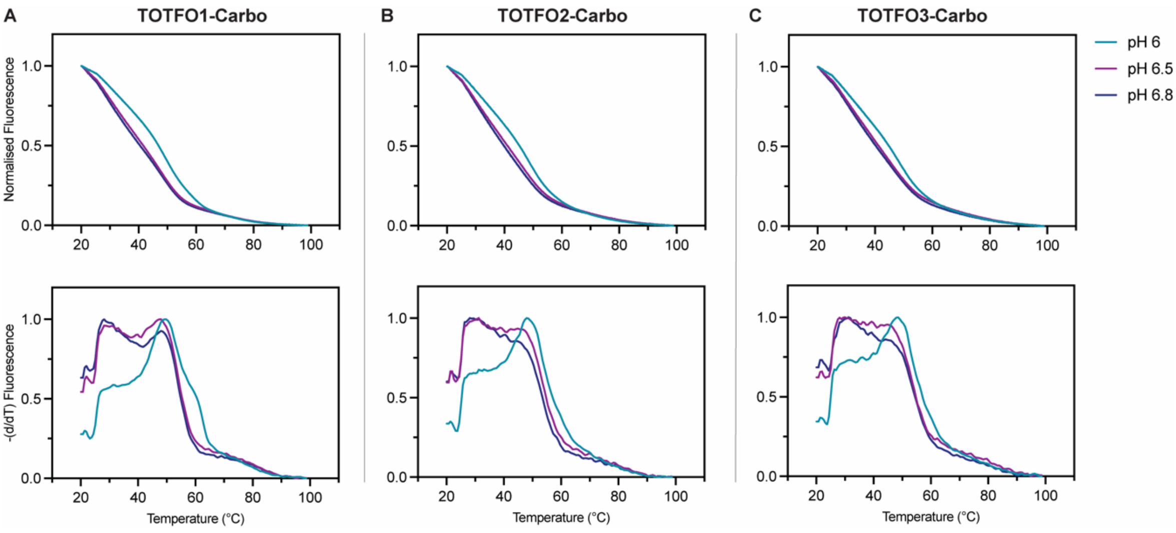

Figure S6. (**Top**) Normalised fluorescence melting curve plot and (**Bottom**) first negative derivative for fluorescence melting for (**A**) TOTFO1-Carbo, (**B**) TOTFO2-Carbo and (**C**) TOTFO3-Carbo. No triplex melting was observed for any hybrids at pH 7.

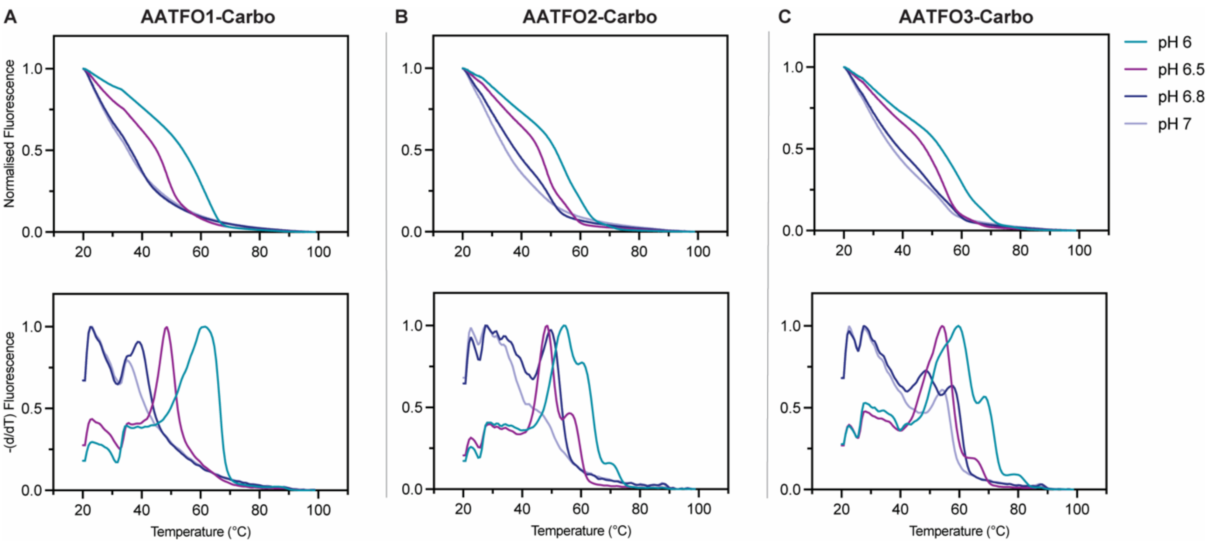

Figure S7. (**Top**) Normalised fluorescence melting curve plot and (**Bottom**) first negative derivative for fluorescence melting for (**A**) AATFO1-Carbo, (**B**) AATFO2-Carbo and (**C**) AATFO3-Carbo.

S3. Pt(II)-TFO Hybrid Click Chemistry Yields

Table S3. **5'-dC** = 5'-(5)octadiynyl-deoxycytidine; **pdU-TO** = 5-(1-propargylamino)-deoxyuridine with TO_{B6} coupled; **Pt-N$_3$-Carbo** = *cis*-[Pt(2-azidopropane-1,3-diamine)(CBDCA)]. Platinum(II)-TFO hybrid yields were calculated with the Beer-Lambert Law using obtained absorbance values after quantification on a NanoDrop ND-1000 UV-Vis Spectrophotometer.

Oligo	Modification	Yield (%)
**TFO1-Carbo**	5'-dC-N$_3$-Pt(II)-Carbo	28.3
**TOTFO1-Carbo**	5'-dC-N$_3$-Pt(II)-Carbo pdU-TO (x1)	43.4
**TOTFO2-Carbo**	5'-dC-N$_3$-Pt(II)-Carbo pdU-TO (x2)	32.7
**TOTFO3-Carbo**	5'-dC-N$_3$-Pt(II)-Carbo pdU-TO (x2)	29.0
**AATFO1-Carbo**	5'-AP-C3-dT-TO, 5'-AP-C3-dT-N$_3$-Pt(II)-Carbo	65.5
**AATFO2-Carbo**	5'-AP-C3-dT-TO, 5'-AP-C3-dT-N$_3$-Pt(II)-Carbo, pdU-TO	55.7
**AATFO3-Carbo**	5'-AP-C3-dT-TO, 5'-AP-C3-dT-N$_3$-Pt(II)-Carbo, pdU-TO (x2)	49.2

S4. Pt(II)-TFO Hybrids PAGE

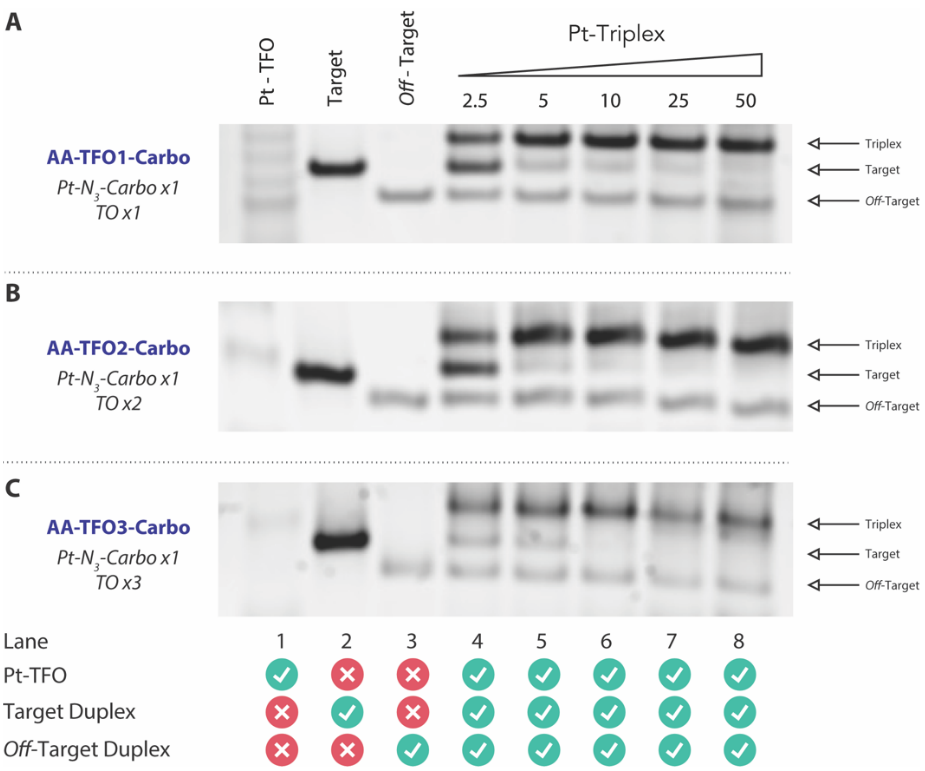

Figure S8. Triplex-crosslink formation for AATFO1-3 Carbo. Target (57 bp, 1 pmol) and off-target (40 bp, 1 pmol) treated with increasing concentrations of Pt(II)-TFO hybrid (2.5-50 eq., lanes 4-8) in triplex buffer. Pt(II)-TFO hybrid only crosslinks with target sequence. No ablation of off-target duplex is observed. PAGE analysis for Pt(II)-TFO hybrids was performed in Tris acetate triplex buffer (10 mM phosphate 150 mM NaCl 2 mM $MgCl_2$, pH 6) at 70 V for 240 mins. Gels were post-stained with SYBR gold.

S5. Pt(II)-TFO Hybrid pH Comparison

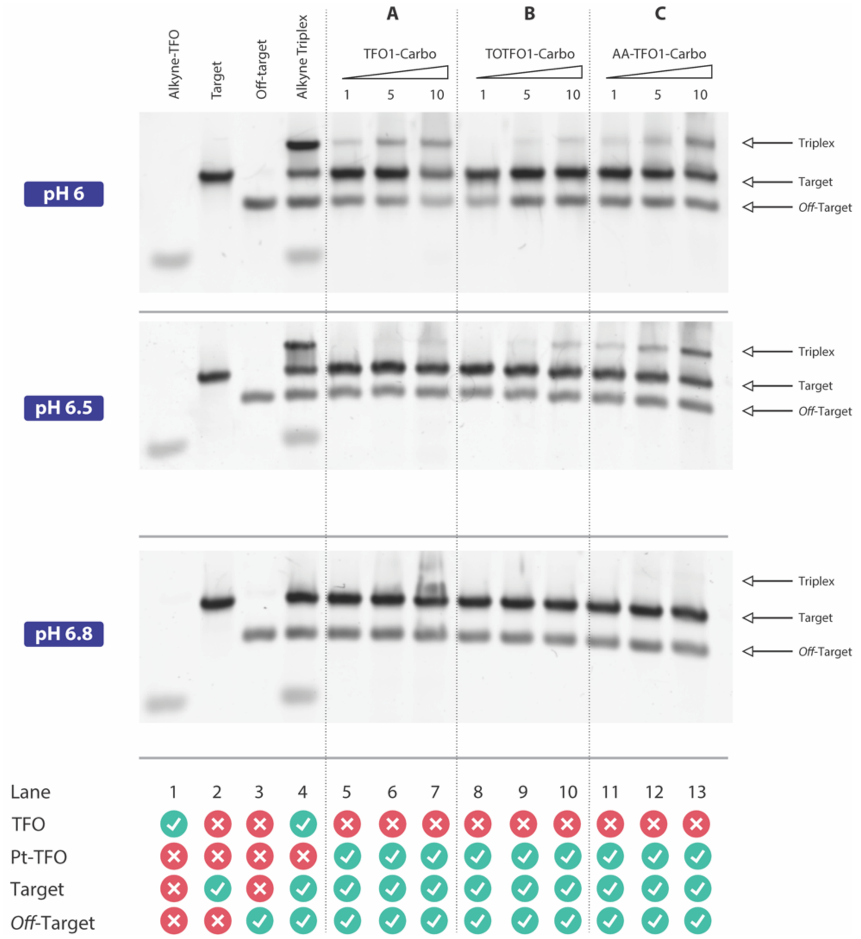

Figure S9. Triplex formation comparison between (**A**) TFO1-Carbo; (**B**) TOTFO1-Carbo; and (**C**) AATFO1-Carbo.Target (57, 1 pmol) and off-target (40 bp, 1 pmol) duplex incubated with increasing concentrations of Pt(II)-TFO hybrid (1, 5 and 10 eq.). Lane 4 with alkyne triplex formation acts as reference for Pt(II) crosslink mediated triplex formation. AATFO1-Carbo forms triplex up to 10 eq. at all pH measurements in comparison to TOTFO1-Carbo and TFO1-Carbo. PAGE analysis for Pt(II)-TFO hybrids was performed in Tris acetate triplex buffer (10 mM phosphate 150 mM NaCl 2 mM $MgCl_2$, pH 6, 6.5 or 6.8) at 70 V for 240 mins. Gels were post-stained with SYBR gold.

S6. Fluorescent Triplex Formation

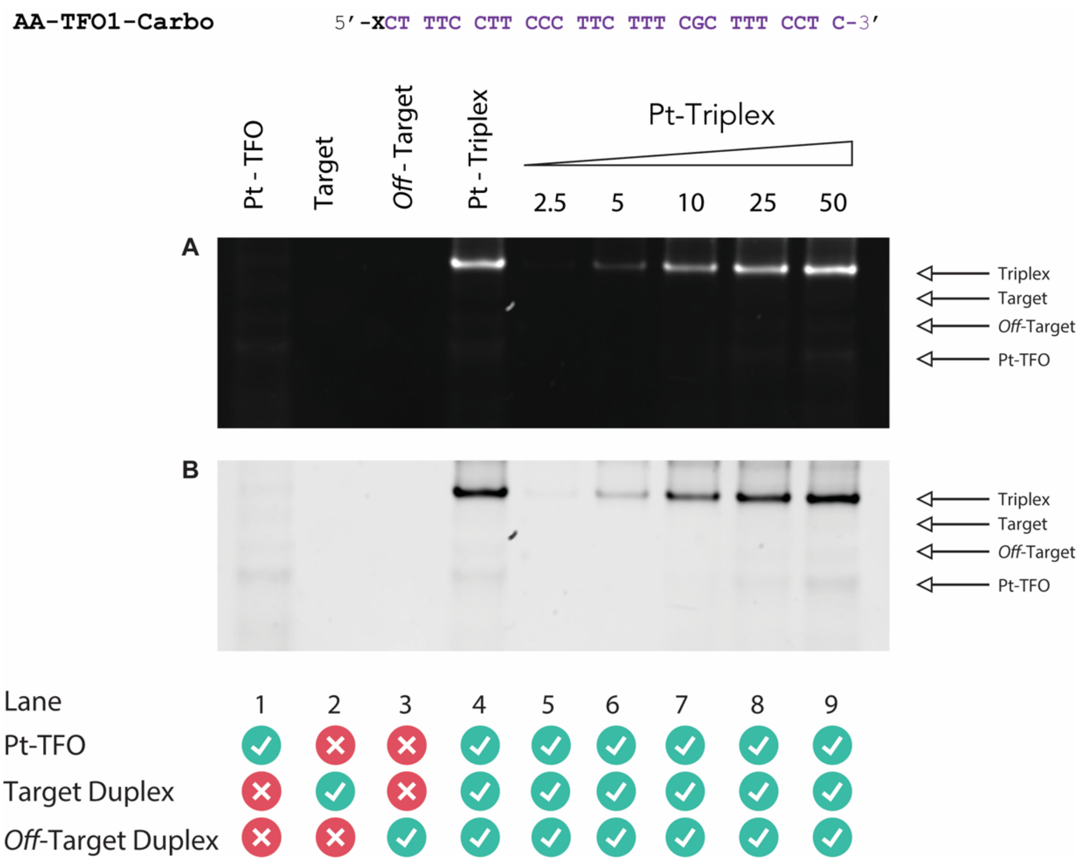

Figure S10. PAGE analysis of AATFO1-Carbo. Figure shown is PAGE displayed in manuscript **Figure 4C** without any additional post-staining. PAGE gel is visualised using standard filter for SYBR gold imaging. (**Top, A**) and (**Bottom, B**) represent different contrasts of the same gel to identify increased TO_{B6} intercalation with triplex formation as Pt-hybrid equivalents increases (2.5 – 50 eq., lanes 5-9). PAGE analysis for Pt(II)-TFO hybrids was performed in Tris acetate triplex buffer (10 mM phosphate 150 mM NaCl 2 mM $MgCl_2$, pH 6) at 70 V for 240 mins.

S7. NaCN Crosslink Reversal PAGE

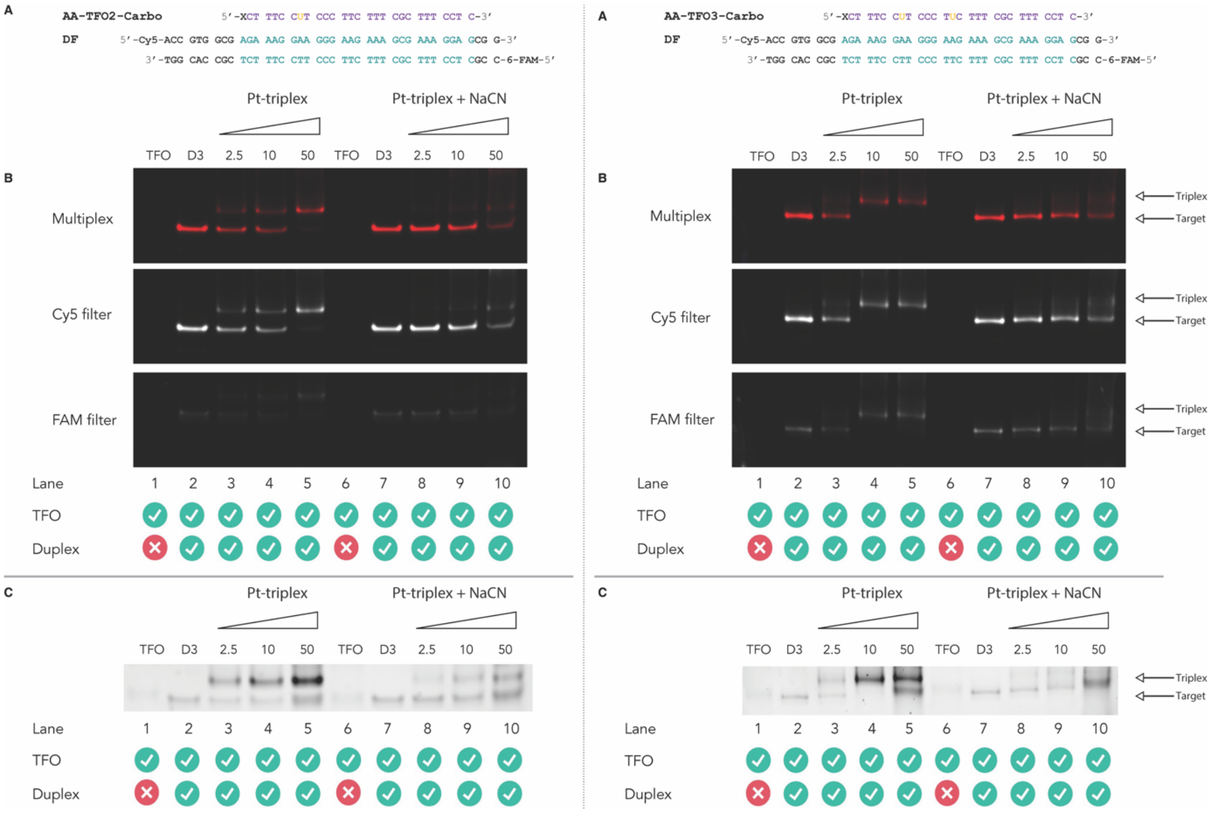

Figure S11. (**Left, A**) AATFO2-Carbo hybrid (28 nt) and fluorescently labelled duplex target **DF** (40 bp). **B.** DF treated with increasing concentrations of Pt(II)-TFO hybrid (2.5, 10 and 50 eq., lanes 3-5) and DF treated with increasing concentrations of Pt(II)-TFO hybrid (2.5,10 and 50 eq., lanes 8-10) prior to treatment and incubation with NaCN solution (5,000 eq.). Gel imaged using Cy5 and FAM filters with multiplex overlay. **C.** Gel post-stained with SYBR gold. (**Right, A**) AATFO3-Carbo hybrid (28 nt) and fluorescently labelled duplex target **DF** (40 bp). **B.** and **C.** Gel performed as AATFO2-Carbo. NaCN PAGE analysis for Pt(II)-TFO hybrids was performed in Tris acetate triplex buffer (10 mM phosphate 150 mM NaCl 2 mM $MgCl_2$, pH 6) at 70 V for 240 mins.
